# Improving the Antibacterial Properties of Dental Bonding Materials Loaded with Silver Compounds

**DOI:** 10.3390/antibiotics12121721

**Published:** 2023-12-13

**Authors:** Elena Ferrando-Magraner, Verónica García-Sanz, Carlos Bellot-Arcís, Anna Marín-Gozalbo, Luís Cabedo-Mas, Gladys Mínguez-Vega, Vanessa Paredes-Gallardo

**Affiliations:** 1 Orthodontics Teaching Unit, Department of Stomatology, Faculty of Medicine and Dentistry, University of Valencia, 46010 Valencia, Spain; elenaferrandomagraner@gmail.com (E.F.-M.); carlos.bellot@uv.es (C.B.-A.); vanessa.paredes@uv.es (V.P.-G.); 2 Polymers and Advanced Materials Group (PIMA), Universitat Jaume I (UJI), Av. De Vicent Sos Baynat s/n, 12071 Castellón, Spain; anmarin@uji.es (A.M.-G.); lcabedo@uji.es (L.C.-M.); 3 GROC·UJI, Institute of New Imaging Technologies, Universitat Jaume I, Av. De Vicent Sos Baynat s/n, 12071 Castellón, Spain; gminguez@uji.es

**Keywords:** nanoparticles, nanotechnology, orthodontic adhesive, dental bonding materials, antibacterial, antimicrobial, antibiofilm, anti-infective, bactericidal, bacteriostatic

## Abstract

Biofilm accumulation, the appearance of white spot lesions and the development of secondary caries are the main complications in orthodontic patients. A promising approach to fight this situation is the development of adhesive cements with improved antibacterial properties. The aim of the present study was to evaluate the possibility of improving the antibacterial properties of glass ionomer cements by incorporating different types of antimicrobial compounds without altering their physical and mechanical properties. Different concentrations of silver carbonate (SC) and an inorganic glass with encapsulated silver were added to the glass ionomer cement, as well as chitosan, to achieve synergistic antibacterial activity. Variations in the antibacterial capacity were evaluated using the agar diffusion test; the potential alteration of the physical and mechanical properties of the material was analyzed by the shear bond strength test. SEM characterization and colorimetric evaluation were also conducted. Samples of SC up to 1% and inorganic glass with encapsulated silver up to 5% showed significant improvement in their antibacterial ability without compromising shear strength. The highest antimicrobial activity was observed for *Lactobacillus acidophilus*, with inhibition zones of 3.8 and 3.1 mm for SC and inorganic glass, respectively. The characterization of the samples did not detect any major structural changes between the different samples. The only group that underwent a noticeable color change was the group with SC. The results show that the incorporation of silver carbonate and inorganic glass with encapsulated silver provided the glass ionomer cement with an antibacterial capacity without compromising the bond strength and without modifying the structure of the material.

## 1. Introduction

Currently, adhesive cements are essential materials in dentistry and are used for the adhesion of crowns or prosthetic abutments and for the fixation of orthodontic devices [1].

However, the placement of fixed orthodontic appliances involves a problem almost inherent to their use: brackets, metal ligatures, archwires, and elastomeric ligatures favor the accumulation and prolonged retention of plaque on the enamel surfaces adjacent to orthodontic appliances, which are difficult to clean with toothbrushing [2]. Orthodontic devices hinder the maintenance of proper oral hygiene, increase biofilm accumulation and lead to high levels of cariogenic bacteria [1,3,4,5]. These cariogenic bacteria produce organic acids and trigger enamel demineralization, of which the appearance of white spot lesions (WSLs) is the first sign of enamel demineralization [6,7,8,9].

The appearance of WSLs around brackets and orthodontic devices is one of the main complications in patients undergoing fixed orthodontic treatment [10]. The development of new white spot lesions in patients during fixed orthodontic treatment has a reported incidence of 45.8%. However, the prevalence of such lesions in patients undergoing orthodontic treatment is 68.4% [11,12]. Factors that increase the incidence of WSL are being of a young age at the beginning of treatment, poor oral hygiene, male sex, and long treatment [12].

The development of caries complicates the course of treatment and reveals the need to control oral biofilms during orthodontic treatment [13]. Dental caries is a multifactorial disease that is closely related to the microbial flora of the oral cavity. Orthodontic treatment with fixed appliances leads to increased plaque accumulation and high levels of *Streptococcus mutans* and *Lactobacillus acidophilus*, which are considered the main pathogens of dental caries [5]. *S. mutans*, the pathogen responsible for the development of caries lesions, is an acidogenic bacterium that, when exposed to fermentable carbohydrates present in the oral biofilm, can produce organic acids and generate enamel demineralization [2,4,5,8,14]. *Lactobacilli* are Gram-positive, facultative anaerobic bacteria that are related to the progression of caries lesions. Thus, *Lactobacillus* does not directly cause dental caries, but its metabolic byproduct, lactic acid, plays an important role in the progression of caries lesions [5,14].

Preventing enamel demineralization and WSL formation is one of the main challenges for clinicians, as such lesions are unsightly and potentially irreversible [7]. Therefore, different methods have been proposed over time to prevent and combat them: oral hygiene improvements, diet modifications, and topical fluoride applications. However, all these methods depend on the patient’s cooperation and compliance and are therefore unreliable [10]. Consequently, preventive measures that do not require patient cooperation may be more effective [10].

Bacteria are the main culprits responsible for the shortened service life of restorations and for the appearance of WSLs [1]. Therefore, one of the most reliable methods to prevent enamel demineralization and decrease the incidence of secondary caries is the use of bonding materials that suppress bacterial activity at the tooth–restoration interface [1,15].

Despite bacterial colonization and, consequently, secondary caries and WSL, dental bonding materials play an irreplaceable role in the field of adhesive dentistry, providing multiple advantages, such as good manageability, excellent esthetics, and acceptable biocompatibility [16]. Thus, numerous attempts are being made to develop dental bonding materials with good long-term behavior and antibacterial activity without compromising their mechanical properties [15,16].

Several methods have been described to improve the antibacterial capacity of dental bonding materials. Much of the literature has focused on the use of materials with fluorides in their composition, such as glass ionomer cements (GICs), or on the incorporation of fluorides into the structure of adhesive cements as a mechanism to prevent the appearance of WSLs. However, GICs used for the fixation of orthodontic devices, despite incorporating fluorides in their composition, present insufficient antibacterial effects to prevent biofilm formation and affect cell viability, thus not reducing the incidence of WSL [17]. While some studies suggest that GICs offer cariostatic activity due to fluoride release, other studies indicate that the degree of fluoride leaching is less than that needed for the development of antimicrobial activity and that antimicrobial effects are not observed after complete curing of the material. Therefore, fluoride release alone cannot be relied upon for effective antibiofilm activity in GICs [18].

One of the most important advances in the field of dental materials is the application of nanotechnology [19]. Nanotechnology is an emerging field of research with very diverse and interesting applications in science and technology, especially for the development of new materials. This raises the possibility of creating new materials with enhanced properties that can prevent recurrent caries and prolong the effectiveness of the treatment [20,21,22]. Nanotechnology has become an important area of research in dentistry, focusing mainly on improving the antibacterial and mechanical properties of dental materials [23,24].

Many studies have analyzed the different available methods to prevent the appearance of white spot lesions around orthodontic appliances. However, this conglomerate of literature has important limitations: most of the studies require the patient’s collaboration for the implementation of measures to reduce bacterial concentration or employ techniques to enhance the antibacterial properties but do not evaluate the possible influence of such modifications on the physical or mechanical properties of the material.

Consequently, the objectives of the present study were to evaluate the antibacterial properties of glass ionomer cement after the incorporation of silver compounds (silver glass, silver carbonate), as well as chitosan (a non-silver based compound with known antimicrobial capacity), used together with the silver compounds in order to obtain synergistic antibacterial activity [17]; to study the differences in terms of the antibacterial properties between the different groups of modified materials; to analyze whether the incorporation of antimicrobial compounds leads to an improvement in the mechanical properties of the material; to evaluate the possible colorimetric variation of the material; and to analyze the changes in the structure of the material.

## 2. Materials and Methods

A comparative in vitro study was carried out using biological samples (human teeth). The study was approved by the Experimental Research Ethics Committee of the University of Valencia (H1518676960681), complying with the fundamental principles established in the Declaration of Helsinki, the Council of Europe Convention on Human Rights, and the requirements established in the Spanish legislation in the field of biomedical research, personal data protection, and bioethics.

### 2.1. Materials Used

The material used was the commercially available glass ionomer cement Ketac™ Cem Easymix (3M™ Deutschland GmbH, Neuss—Germany). Ketac™ Cem is a self-curing, radiopaque glass ionomer cement consisting of a powder (glass powder, polycarboxylic acid, and pigments) and a liquid (water, tartaric acid, and preservatives). It is indicated for the bonding of metal, metal–ceramic, or metal–resin inlays and onlays, for the cementation of metal or ceramic posts and pins, and for the fixation of orthodontic devices. For its preparation, a ratio of 3.8 portions of powder to 1 portion of liquid was used, following the manufacturer’s indications.

The glass ionomer cement was modified by incorporating three different types of compounds:

- Silver carbonate (SC) (Merck Life Science S.L., Madrid, Spain. Batch number: BCCB4406).
- Low molecular weight chitosan (CH) (50,000–190,000 Da) (Merck Life Science S.L., Madrid, Spain. Batch number: BCCD9853).
- Inorganic glass powder with encapsulated silver (Asepticae 013Ag) (Encapsulae S. L., Castelló, Spain).

### 2.2. Sample Preparation

Circular samples (5 mm × 2 mm) used for the characterization, colorimetric, and microbiological studies were prepared using unmodified GIC and GIC modified with the different compounds. All the samples were prepared using a silicone matrix of 200 mm × 100 mm × 2 mm with preprepared circular perforations of 5 mm in diameter and two glass slides. The glass ionomer cement was prepared according to the manufacturer’s instructions and modified by incorporating silver carbonate, inorganic powder with encapsulated silver, or chitosan in different weight proportions. Unmodified GIC was used as a control. Once the material was prepared, it was compacted into the matrix located on top of a glass slide, covered with the other glass slide, and left to dry for 30 min at 20–25 °C. The samples were then removed from the mold, stored in a dark room at 37 °C for 24 h, polished using a commercial polishing system (Sof-Lex™ disc system, 3M™ Espe, St Paul, MN, USA), sterilized by drizzling them with 70% (*v*/*v*) ethanol, and, finally, rinsed with a saline solution to remove any residue from the polishing or disinfection process. For the shear test, the last phase of sample preparation was different from that detailed above.

The samples were prepared as follows:

- Control group: unmodified GIC powder and liquid were mixed according to the manufacturer’s instructions with a powder/liquid (P/L) ratio of 3.8/1.
- GIC + SC (0.5%, 1%, 2%): the GIC powder was modified by incorporating 0.5, 1, and 2% SC (*w*/*w*). Both powders were mixed and stirred, and, finally, the SC-modified GIC powder was mixed with the unmodified GIC liquid following the manufacturer’s instructions.
- GIC + inorganic powder with encapsulated silver (1%, 2.5%, 5%): the GIC powder was partially replaced with 1, 2.5, and 5% silver glass (*w*/*w*). Both powders were mixed and stirred, and, finally, the modified GIC powder was mixed with the unmodified GIC liquid according to the manufacturer’s instructions.
- GIC + chitosan (2.5%, 5%, 7.5%, 10%): a dispersion of 2% CH (*w*/*v*) in 0.3 N acetic acid was prepared and mixed with the commercial GIC liquid in different proportions to achieve the concentrations above. Finally, the unmodified GIC powder was mixed with the CH-modified GIC liquid following the manufacturer’s instructions.
- GIC + chitosan + SC: the CH was incorporated into the liquid part of the GIC in different concentrations, as indicated in the previous group (5% and 10%), and SC was added to the powder part of the GIC (0.5% and 1%). Both the modified liquids and powders were then used to prepare the samples.

Table 1 shows the composition of the control group and each of the experimental groups, as well as the nomenclature used for each group.

### 2.3. Evaluation of Antibacterial Properties

#### 2.3.1. Microorganisms and Growth Conditions

*Lactobacillus acidophilus* (CECT 903) and *Streptococcus mutans* (CECT 479) strains were obtained from the Spanish Type Culture Collection (University of Valencia, Burjassot, Valencia, Spain). MRS and brain infusion (BHI) culture media were used for the microbiological antibacterial activity assays (Scharlab S. L., Barcelona, Spain). For culture activation, from previously inoculated MRS or BHI agar plates, several isolated colonies were selected and, using an inoculation loop, transferred to tubes containing 5 mL of the corresponding broth. The tubes were incubated overnight at 37 °C under aerobic conditions for *S. mutans*. The incubation of *L. acidophilus* was carried out in anaerobic conditions using an anaerobic jar and anaerobic gas-generating sachets (AnareroGenTM; Oxoid Ltd., Basingstoke, England). The bacterial growth was determined by measuring the optical density of the liquid cultures at 600 nm using a spectrophotometer (V-1100, J. P. Selecta, Abrera, Barcelona, Spain). The inoculum concentration of the culture was adjusted to 10^7^ colony-forming units (CFU)/mL.

#### 2.3.2. Minimum Bactericidal Concentration

The minimum bactericidal concentration (MBC) of the silver carbonate (SC) was determined as reported by Andrade [25], with modifications.

Different SC solutions were prepared at concentrations ranging from 2000 to 10 ppm, and 100 µL of each was deposited in 96-well plates. Then, 100 µL of MRS or BHI broth was added, and each well was inoculated with 5 × 10^5^ CFU of *L. acidophilus* or *S. mutans*.

The positive control (C1) consisted of broth plus bacterial inoculum, while the negative controls were broth only (C2) and broth plus inoculum and 100 µL of 0.2% (*w*/*v*) antibiotic (streptomycin sulfate salt, Merck Life Science S.L., Madrid, Spain) (C3). The plates were incubated for 24 h at 37 °C under aerobic or anaerobic conditions depending on the strain. After incubation, serial dilutions of each bacterium and SC solution were made and seeded on solid media to determine bacterial survival. The MBC of SC was the lowest concentration at which no colony growth was observed after 24 h at 37 °C [26].

The experiments were performed in triplicate.

#### 2.3.3. Evaluation of Antibacterial Activity

To avoid cross-contamination, prior to the assays, the discs were sterilized by immersion in 70% (*v*/*v*) ethanol and allowed to dry for 20 min. The antibacterial activity of each material was evaluated by the agar diffusion test, according to Andrade [25].

The agar plates were inoculated by passing a swab in at least 4 directions to ensure a complete distribution of the inoculum on the agar surface. Each disk was placed on the agar using sterile forceps and pressed gently to ensure full contact between the material and the medium. The plates were incubated at 37 °C for 24 h, and the diameter of the inhibition zone around each disk was measured.

A positive control consisting of a paper disk impregnated with 10 µL of streptomycin sulfate salt (0.2%, *w*/*v*) was used.

Three replicates of each material were tested for each microorganism.

### 2.4. Sample Characterization

The microstructure of the materials was examined by scanning electron microscopy (SEM) using a JEOL 7001F high-resolution field emission microscope (Tokyo, Japan). The samples were coated with a thin layer of platinum prior to observation at an accelerating voltage of 5 kV. The first observations were made at 50–500× magnification to obtain an overview of the structure of the material both in cross-section and on the surface, and then observations were made at higher magnifications (500–10,000×) to analyze in detail the most characteristic aspects of each of the observed structures.

The distribution of the SC in the samples was analyzed by energy dispersive X-ray spectroscopy (EDX).

### 2.5. Evaluation of Adhesion Properties (Shear Bond Strength)

The resistance of the material’s bond to shear strength was tested with human teeth (permanent molars and premolars) without caries, extracted for reasons unrelated to the research in the dental clinic of the University of Valencia. Informed consent was obtained from the patients who donated their teeth. The use of human teeth for this study was approved by the Ethics Committee on Experimental Research of the University of Valencia (H151867696960681).

Forty-five teeth were collected, cleaned, and stored in a saline solution at 34 °C for a maximum of 60 days from the time of extraction. The storage water was changed every five days to prevent bacterial growth on the tooth surfaces. These 45 teeth were randomly distributed into 15 groups. For each of the samples, three replicates were tested.

Each tooth was vertically fixed into a working platform using a self-curing acrylic resin, taking the axial axes of the clinical crowns as a reference so that the tooth’s proximal surface was parallel to the force during the shear strength test. To ensure a flat and regularized bonding surface, the proximal surfaces of the teeth were polished using a water-cooled high-speed diamond bur (Gebr. Brasseler GmbH & Co. KG, Lemgo, Germany). Subsequently, the surfaces were washed with distilled water and then air-dried.

The samples of control glass ionomer cement and modified glass ionomer cement were prepared as detailed above and immediately fixed on the most central area of the middle third of the anatomical crowns using a silicone mold.

After the bonding procedures, the bonded tooth–cement assemblies were kept at room temperature for 60 min and then incubated in distilled water at 37 °C for 24 h prior to mechanical testing.

The bond strength to shear forces was determined using a Shimadzu universal testing press (Japan). A chisel-type attachment was incorporated at the end of this computer-controlled testing machine, which was oriented to coincide with the top of the specimen adhered to the proximal surface of the tooth. An occluso-gingival load was applied at a crosshead speed of 1 mm/min, producing a shear force at the interface between the tooth and the specimen until the bond failed and the force at fracture was recorded.

### 2.6. Evaluation of the Colorimetric Properties

A color evaluation was performed using the VITA Easyshade^®^V spectrophotometer (VITA Zahnfabrik; Bad Säckingen, Germany), which uses the color space L*a*b* of the Commission Internationale de l’Eclairage (CIE L*a*b*) in comparison with a white calibration control. The color of the samples was evaluated after storage in a dark room at 37 °C for 24 h and polishing. The color differences resulting from the incorporation of different compounds were expressed as ΔE*, where L* represents the difference between light (L* = 100) and total darkness (L* = 0), a* represents the difference between red (a*+) and green (a*−), and b* represents the difference between yellow (b*+) and blue (b*−).

### 2.7. Statistical Analysis

A statistical analysis of the results was performed using Statgraphics Centurion XVI version 16.1.17 (Manugistics Corp., Rockville, MD, USA).

The differences between the different samples in both the diameter of the bacterial growth inhibition zone and the shear strength resistance were statistically analyzed. One-way ANOVA was used; the inhibition zone diameter and shear bond strength were considered the dependent variables, and the type of material was considered a factor in both cases. *p* values lower than 0.05 were considered statistically significant. All the data are expressed as the means ± standard deviations.

## 3. Results

### 3.1. Antibacterial Properties

#### 3.1.1. Minimum Bactericidal Concentration of Silver Carbonate

Table 2 shows the results regarding the minimum bactericidal concentration (MBC) of SC against the two bacterial species evaluated, as well as the percentage of reduction achieved with each of the different concentrations of the compound. The MBC of silver carbonate (SC) was 50 ppm for *L. acidophilus* and 1000 ppm for *S. mutans*. *L. acidophilus* is much more sensitive to this antimicrobial than *S. mutans*, so a comparable inhibition can be achieved with a lower amount of SC.

#### 3.1.2. Antibacterial Activity

**Figure 1:**
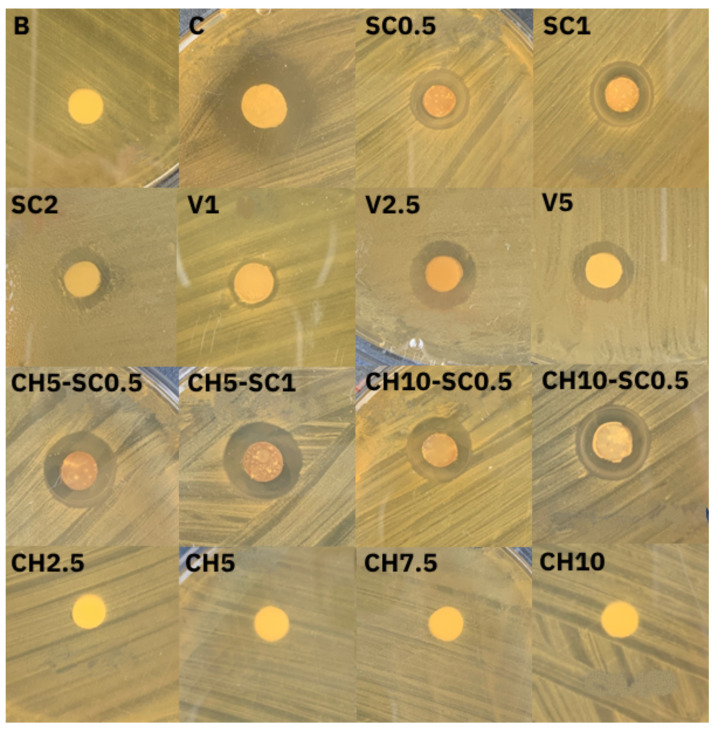
Inhibition zones obtained in culture plates inoculated with *L. acidophilus*. B: nonmodified glass ionomer cement (GIC); C: positive control (cellulose impregnated with antibiotic); SC0.5: GIC modified with 0.5% SC; SC1: GIC modified with 1% SC; SC2: GIC modified with 2% SC; V1: GIC modified with 1% inorganic glass with encapsulated silver; V2.5: GIC modified with 2.5% inorganic glass with encapsulated silver; V5: GIC modified with 5% inorganic glass with encapsulated silver; CH2.5: GIC modified with 2.5% chitosan (CH); CH5: GIC modified with 5% CH; CH7.5: GIC modified with 7.5% CH; CH10: GIC modified with 10% CH; CH5-SC0.5: GIC modified with 5% CH and 0.5% SC; CH5-SC1: GIC modified with 5% CH and 1% SC; CH10-SC0.5: GIC modified with 10% CH and 0.5% SC; CH10-SC1: GIC modified with 10% CH and 1% SC.

**Figure 2:**
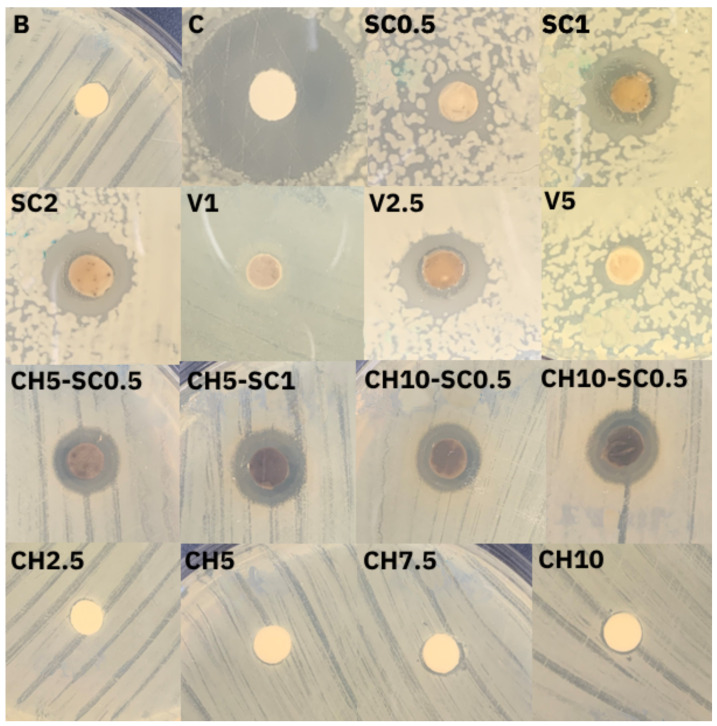
Inhibition zones obtained in culture plates inoculated with *S. mutans*. B: nonmodified glass ionomer cement (GIC); C: positive control (cellulose impregnated with antibiotic); SC0.5: GIC modified with 0.5% SC; SC1: GIC modified with 1% SC; SC2: GIC modified with 2% SC; V1: GIC modified with 1% inorganic glass with encapsulated silver; V2.5: GIC modified with 2.5% inorganic glass with encapsulated silver; V5: GIC modified with 5% inorganic glass with encapsulated silver; CH2.5: GIC modified with 2.5% chitosan (CH); CH5: GIC modified with 5% CH; CH7.5: GIC modified with 7.5% CH; CH10: GIC modified with 10% CH; CH5-SC0.5: GIC modified with 5% CH and 0.5% SC; CH5-SC1: GIC modified with 5% CH and 1% SC; CH10-SC0.5: GIC modified with 10% CH and 0.5% SC; CH10-SC1: GIC modified with 10% CH and 1% SC.

Figure 1 and Figure 2 show representative images of the antibacterial agar diffusion assay and the inhibition zones obtained with the samples when applied on cultured plates. For both bacterial species, the greatest inhibition zones were obtained in the groups with silver carbonate and inorganic glass with encapsulated silver. The samples with chitosan and unmodified control GIC did not show inhibition zones, indicating that neither chitosan nor the base material itself exerted antimicrobial activity against the studied bacteria.

These results show that incorporating a silver-based compound provides the material with a bactericidal capacity and allows it to have an effect beyond the point of application.

For the samples that included the combination of CH and SC, very similar growth inhibition zones were generated, both in appearance and size, to the samples that only incorporated SC.

**Figure 3:**
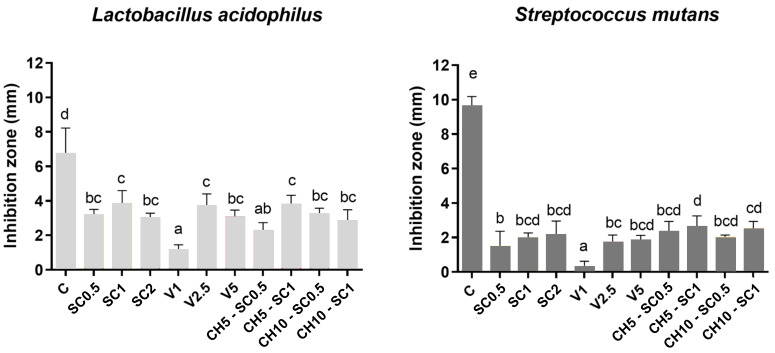
Antibacterial activity of glass ionomer discs modified with silver carbonate, inorganic glass with encapsulated silver, and silver carbonate with chitosan against *S. mutans* and *L. acidophilus*, determined by the direct contact technique. C: control consisting of a cellulose disk impregnated with antibiotic. Superscripts: groups with different letters (a–e) show statistically significant differences using the LSD test (*p* < 0.005).

Regarding the measurement of the inhibition zones for both bacterial species (Figure 3), it was observed that, in general, the levels of inhibition achieved for *L. acidophilus* were higher than those for *S. mutans*, in agreement with the results of MBC and demonstrating the higher resistance of the latter. Only the values relating to the samples that exhibited a certain bactericidal capacity were included in this analysis, thus excluding the data relating to the control samples and the samples incorporating only chitosan.

For *L. acidophilus*, the highest levels of inhibition were achieved with SC at 1%, with a value of 3.8 ± 0.8 mm; however, a statistical analysis revealed that this value was not significantly higher (*p* > 0.005) than those obtained with 2 and 0.5%. A similar trend was observed for *S. mutans*, with diameters of 2.1 ± 0.9 mm and 2.0 ± 0.3 mm for the 2 and 1% concentrations, respectively, but with the differences not being statistically significant compared with the 0.5% formulation.

Regarding the SC samples, concentrations of 0.5 and 1% SC were chosen to be combined with 5 and 10% CH. For both bacterial species, the presence of chitosan did not result in larger inhibition zones since no statistically significant differences (*p* > 0.005) were observed in any case.

The addition of inorganic glass with encapsulated silver at 1% resulted in significantly smaller inhibition diameters (*p* < 0.005) than the other two concentrations studied for this material. No differences were observed between the 2.5 and 5% concentrations, with values of 3.7 ± 0.7 mm and 3.1 ± 0.4 mm for *L. acidophilus* and 1.7 ± 0.4 and 1.8 ± 0.4 mm for *S. mutans*.

### 3.2. Sample Characterization

**Figure 4:**
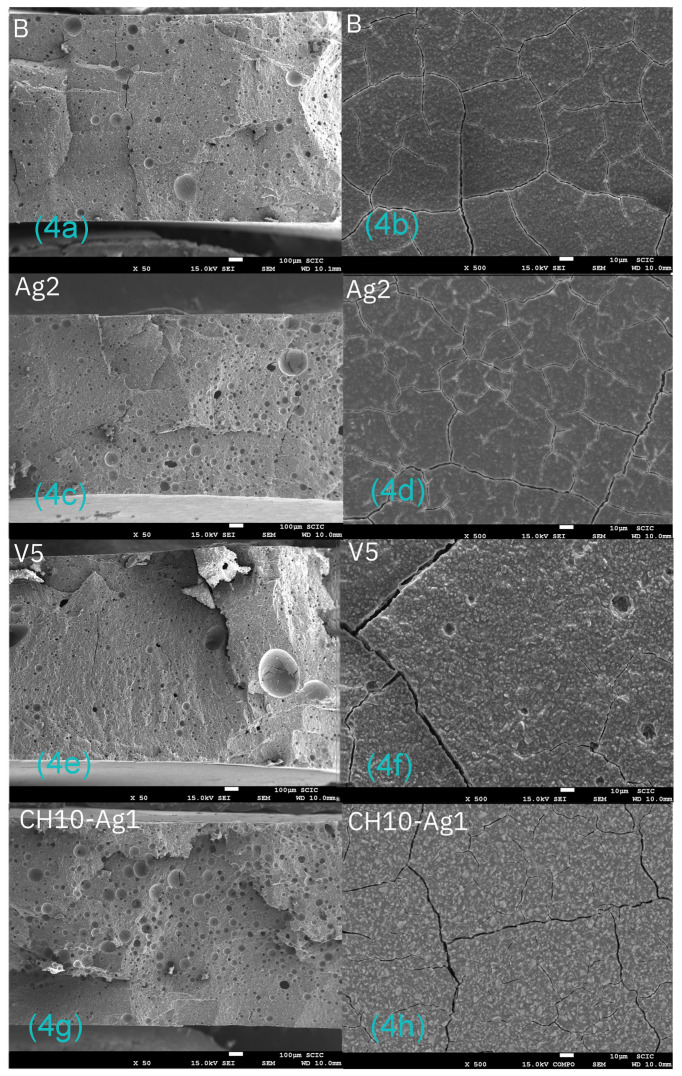
(**a**–**h**) Images obtained by electron microscopy of the cross-section (left side) and surface (right side) of some of the studied materials. (**a**,**b**):control sample. (**c**,**d**) GIC sample with 2% SC. (**e**,**f**) GIC sample with 5% inorganic glass with encapsulated silver. (**g**,**h**) GIC sample with 10% CH—1% SC. Images obtained at 50–500× magnification.

Figure 4a–h shows representative images of samples obtained by scanning electron microscopy at 50–500× magnification; the images on the left side (a, c, e, g) belong to the cross-section, and the images on the right side (b, d, f, h) belong to the surface.

No considerable differences were found between the samples belonging to the different groups, so the incorporation of silver carbonate, chitosan, inorganic glass with encapsulated silver, or the combination of chitosan and silver carbonate did not produce major changes in the structure of the original material.

Only small changes could be observed: the incorporation of 5% inorganic glass with encapsulated silver provided a slight increase in surface roughness (sample V5—Figure 4e), and the samples with 10% chitosan plus 1% silver carbonate showed a clear increase in porosity in the cross-section image (sample CH10–SC1—Figure 4g).

**Figure 5:**
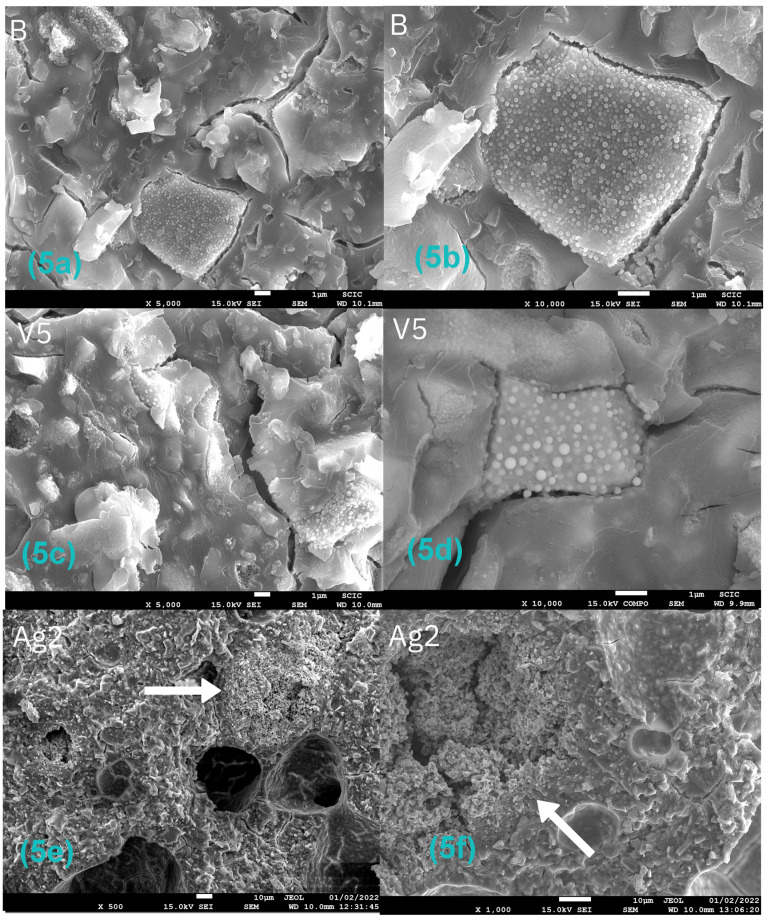
(**a**–**f**) Images obtained by scanning electron microscopy of the cross-section of some of the studied materials. Arrows indicate silver carbonate aggregates. (**a**,**b**) control sample. (**c**,**d**) GIC sample with 5% inorganic glass with encapsulated silver. (**e**,**f**) GIC sample with 2% SC. Images obtained at 500–10,000× magnification.

**Figure 6:**
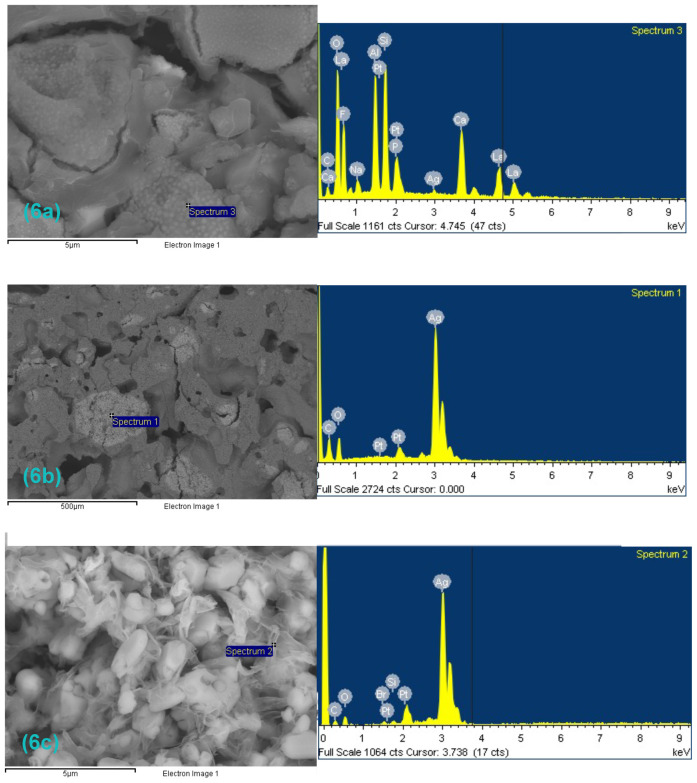
(**a**–**c**) Spectrum obtained from energy dispersive X-ray spectroscopy microanalysis of GIC modified with 2% SC. (**a**) aggregates formed by spherical particles; (**b**,**c**) silver carbonate aggregates.

At higher magnifications (500–10,000×) (Figure 5a–f), the presence of large structures formed by clusters of abundant small spherical particles was observed in all the samples (Figure 5a–d), even in the control samples. A microanalysis by energy dispersive X-ray spectroscopy revealed the presence of significant amounts of lanthanum in these structures (Figure 6a).

In Figure 5e,f, examples of large silver carbonate aggregates can be observed with heterogeneous distribution. A microanalysis of these zones confirmed the presence of silver (Figure 6b,c).

### 3.3. Shear Bond Strength

**Figure 7:**
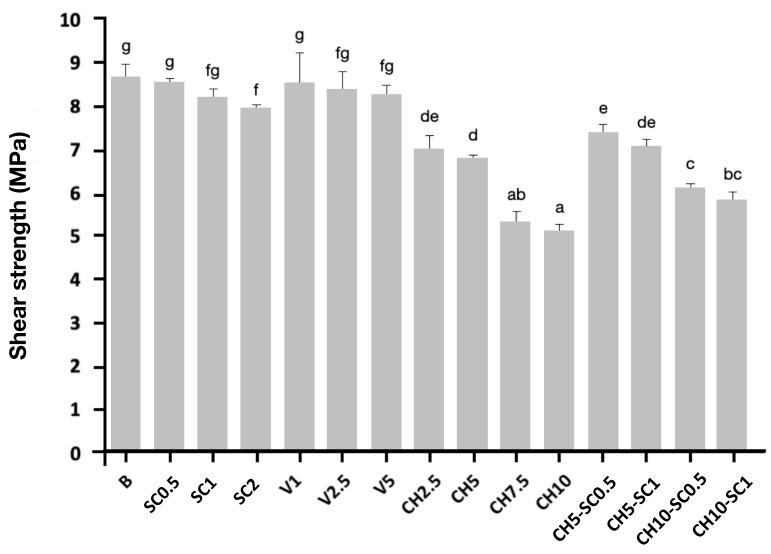
Cement–tooth shear test, expressed in MPa; mean and standard deviation of bond strength data (mean ± sd; *n* = 3). Superscripts: groups with different letters (a–g) show statistically significant differences using the LSD test (*p* < 0.005). In the one-way ANOVA for each bacterial strain, *p* value = 0.000.

The shear bond strength of the control GIC samples and GIC modified with the different compounds bonded to the proximal surface of the teeth is shown in Table 3 and Figure 7; Table 3 shows the force (MPa) needed to fracture the sample at the tooth–cement interface.

The group that presented the highest resistance to shear bond strength was the control GIC (8.62 ± 3.77 MPa), followed by the group of GIC with 0.5% SC (8.49 ± 1.40 MPa), and the group of GICs that incorporated inorganic glass with encapsulated silver at 1% (8.48 ± 7.10 MPa) (Table 3). The differences among the control, silver carbonate, and inorganic glass groups were not statistically significant (*p* > 0.005), so the incorporation of silver carbonate or inorganic glass with encapsulated silver did not worsen the mechanical properties of the GIC.

The shear bond strength values of the different groups identified seven homogeneous groups, with statistically significant differences between them (*p* < 0.05). The multiple range test detected that the samples with the highest shear bond strength were those belonging to groups B, SC0.5, SC1, V1, V2.5, and V5, with no significant differences among them (Table 4).

The addition of 2% SC led to a significant reduction in shear bond strength, being the only group that experienced a significant reduction in shear bond strength with the addition of silver. Therefore, the addition of silver carbonate to the GIC up to 1% and inorganic glass with encapsulated silver up to 5% did not significantly compromise the enamel–cement bond strength.

Chitosan was the component that led to a significant worsening of the adhesion properties of the material. The groups with the greatest decrease in shear bond strength with no significant differences between them were GIC with 10% CH (5.09 ± 1.52 MPa) and GIC with 7.5% CH (5.29 ± 2.65 MPa).

### 3.4. Colorimetric Properties

**Figure 8:**
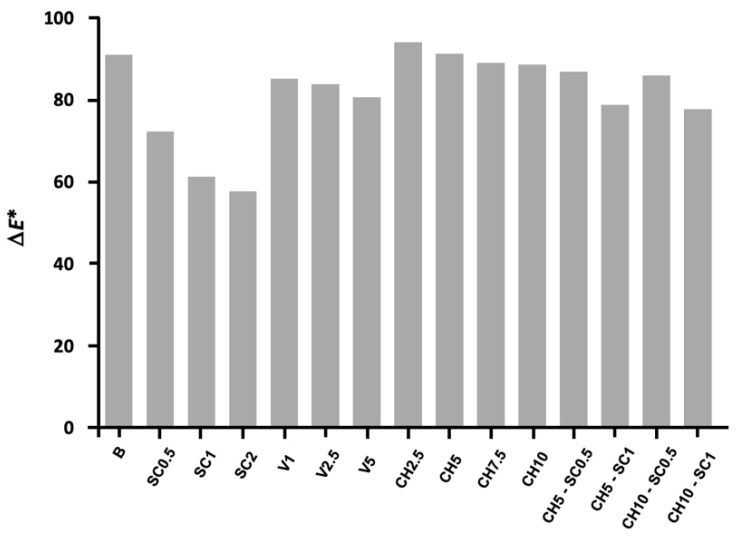
Color determination (ΔE*) for the groups included in this study.

The color determinations (ΔE*) calculated for the modified and unmodified GICs are shown in Figure 8.

The only samples that showed a noticeable color change compared to the unmodified GIC samples were the silver carbonate groups. The GIC samples incorporating chitosan and inorganic glass with encapsulated silver exhibited similar characteristics to the control group.

Regarding the colorimetric characterization of the samples using VITA Easyshade^®^V, the values most similar to the unmodified GIC were recorded for the chitosan groups with L* values equivalent to the control samples. There was an exception with the higher chitosan concentrations (7.5–10%), which showed slightly lower lightness values. The inorganic glass with encapsulated silver groups also presented similar values to the control, with L* values indicating a slightly lower lightness, comparable a* values, and slightly lower b* values, which indicate a more azure hue of these samples in relation to the control samples. However, the incorporation of silver carbonate showed significant color variations: the lightness of these samples was noticeably lower than that of the control samples, although the darkening of these samples did not correlate with the increase in silver carbonate concentrations. The values of a* for these samples indicate a hue with a certain reddish tendency, with the control samples being greener; however, the values of b* show a less yellowish hue than the control samples.

The joint incorporation of silver carbonate and chitosan showed similar values to the control samples in contrast to the values shown when adding silver carbonate alone, despite the silver carbonate concentrations being equivalent in both groups. The joint incorporation of both compounds led to samples with less light than the control sample but not as dark as the samples incorporating silver carbonate only, with a* and b* values similar to the control sample.

Table 5 shows the values in the CIE L*a*b* color space resulting from the colorimetric characterization of the samples using VITA Easyshade^®^V.

## 4. Discussion

The appearance of incipient caries lesions in a relatively short period of time is attributed to the accumulation and prolonged retention of bacterial plaque on the enamel surfaces adjacent to orthodontic appliances. This accumulation of plaque is often difficult to remove by toothbrushing. Although there have been numerous attempts to develop preventive approaches to minimize the risk of caries in patients with fixed orthodontic appliances, patient compliance is a limiting factor in achieving a successful outcome over time, so alternatives that do not depend on patient cooperation are needed. A promising alternative is the development of adhesive cements with antibacterial properties, cements that prevent bacterial adhesion and, thus, reduce the consequent failures associated with fixed orthodontic treatment. Nanotechnology represents an area in which research is currently focused. The use of nanoparticles and nanostructured composites makes it possible to create new materials with superior properties to those traditionally used.

In the present investigation, the incorporation of silver carbonate, encapsulated silver inorganic glass, and chitosan was proposed for different reasons. On the one hand, silver has very good biocompatibility and low toxicity to human cells, presents long-term antibacterial power, and causes less bacterial resistance than antibiotics [21]. Chitosan, on the other hand, in addition to its unique biological characteristics, such as biocompatibility and mucosal adhesion, presents a broad spectrum of antibacterial properties against Gram-positive and Gram-negative bacteria [17,27]. Regarding the inorganic glass with encapsulated silver, it was thought that its incorporation could provide an improvement in the antibacterial activity of GIC without generating a great structural modification of the material. The solid part of the GIC is partially replaced by this compound, but the glass composition is similar, although in this case, it incorporates encapsulated silver. This is a totally innovative compound in this research area since it is a compound provided by a company (Encapsulae S.L., Castellón, Spain) that is accustomed to generating antibacterial coatings on surface materials.

In contrast to previous investigations that also incorporated silver salts into glass ionomer cement with the aim of improving its antibacterial properties, as is the case for Paiva [28], which incorporates SC, in the present work, we opted for the incorporation of silver carbonate. This decision was based on the solubility of the different compounds; silver carbonate has a lower solubility (0.0032 g/100 mL of water) than other silver salts, such as silver nitrate (245 g/100 mL of water) or silver citrate (0.029 g/100 mL of water). A compound with a lower solubility was needed so that, in the process of sample preparation and disinfection, this silver salt would not be released, and its effect was lost in the long term. A compound that is not very soluble, such as silver carbonate, allows for a more controlled release of silver, avoiding the loss of all its effect in the first phases during the handling of the material and the first moments of its introduction into the oral cavity. In addition, the fact that this silver salt is more soluble in acidic environments makes it more favorable in cases of biofilm accumulation and proliferation of cariogenic bacteria.

The results of the present study show that the best results in terms of antibacterial activity were obtained by incorporating silver into the GIC (SC0.5, SC1, Ag2, V2.5, V5). A significant improvement in antibacterial properties was found both qualitatively (Figure 1) and quantitatively (Figure 3), coinciding with other previously published research [10,18,28].

The incorporation of inorganic glass with encapsulated silver (V2.5 and V5) resulted in inhibition values significantly equal to those obtained by incorporating silver carbonate (SC0.5, SC1, SC2), with probably much lower silver values. In the GIC samples with SC, the percentage of the antibacterial compound incorporated into the cement corresponds completely to the silver salt, and in the samples with glass, the percentage of silver is found as silver particles encapsulated within this inorganic glass. This fact could be due to the smaller size of the encapsulated silver particle, which allows a larger contact surface. The smaller particle size would favor the distribution of the antimicrobial agent in the material so that the silver would be more homogeneously distributed, contrary to what happens with silver carbonate, which tends to form aggregates (Figure 5e,f) and would therefore be more available to exert its effect [29].

Regarding the incorporation of chitosan into the cement, the results of the antibacterial test did not show a significant antibacterial effect, in contrast with previous investigations in which a clear antimicrobial effect of chitosan was demonstrated when incorporated into the same dental bonding material [17,27,30]. In this regard, it is worth mentioning that in a pilot investigation carried out prior to this study, other chitosan incorporation strategies were analyzed: chitosan was incorporated both by direct mixing with the GIC powder and by dissolving it in the GIC liquid [17,27,30]. However, in all cases, the results were similar, and an antibacterial effect could not be detected using the same concentrations and the same mechanism of incorporation as previously published studies. Brahim [27] observed that incorporation of chitosan into the polyacrylic acid liquid of GIC in proportions of 5–10% improved the antibacterial properties of GIC against *S. mutans* without adversely affecting its adhesion properties. On the other hand, Debnath [18] corroborated the results of previous research and found that modification of the liquid phase of GIC with 10% CH significantly improved the antibacterial properties, as well as its adhesion. The hypothesis that could explain such disparity between the results would be a difference in the characteristics of the material used. This difference might be attributed to a final lower CH concentration in our materials in comparison to those tested in the aforementioned studies or even to differences in its molecular weight, which is known to be related to its antimicrobial capacity. In the present study, low molecular weight chitosan was used to facilitate its release and ability to act, although no specific analysis was performed to ascertain the exact molecular size of the compound. However, the mentioned studies did not mention the type or characteristics of the CH used.

Although we observed that the samples incorporating chitosan and the control samples generated a slight inhibition of bacterial growth on the contact surface, the presence of such an area was not considered a real inhibition of bacterial growth. The inhibition surface was the same in both cases and corresponded to the exact area of contact of the sample with the plate, which suggests that a bactericidal effect had been produced in that area by direct contact and that this effect was achieved by the material itself and not attributable to the chitosan, since the surface was the same in both cases. This antibacterial capacity of GIC alone is detailed both in the product specifications [31] and in different publications [32,33]. However, in the present investigation, it was not possible to detect a clear bactericidal effect of the control material, as occurs with the silver carbonate or inorganic glass samples with encapsulated silver, only an inhibition of bacterial growth in the contact zone (Figure 2). This would suggest that in these cases, the bactericidal effect occurs by direct contact, while when silver compounds are incorporated into the GIC structure, diffusion of the antimicrobial occurs, which allows for greater bactericidal power to be achieved. Most studies published in the literature agree that conventional glass ionomer cement does not show a real antibacterial effect on its own [17,27,30].

All the groups exhibited significantly higher shear strength results than the control material, with all of them slightly above the force range of 5.9–7.8 MPa recommended by Reynolds [34]: B (8.62 MPa), SC0.5 (8.49 MPa), SC1 (8.14 MPa), V1 (8.48 MPa), V2.5 (8.33 MPa), V5 (8.22 MPa). The incorporation of silver carbonate up to 1% and the incorporation of inorganic glass with encapsulated silver up to 5% into the glass ionomer cement did not significantly compromise the mechanical properties of the material. If these results are added to those obtained by bactericidal tests, it can be affirmed that the incorporation of silver carbonate and inorganic glass with encapsulated silver allows for a superior antimicrobial effect without modifying the structural characteristics of the material in terms of mechanical properties. Further experimental analysis would be useful to achieve a more detailed result. However, the addition of chitosan at 7.5% and 10% produced a reduction in the shear strength of the material. In addition, characterization of the CH10-SC1 samples by scanning electron microscopy detected a clear increase in the porosity of the material (Figure 4g,h), which could explain this worsening of the mechanical properties.

The characterization study showed the presence of significant amounts of lanthanum in all the samples detected by energy dispersive X-ray spectroscopy microanalysis. Such presence was considered a characteristic of the base material since it was present in all the samples; however, no study has previously reported the presence of this compound in the composition of glass ionomer cement. Lanthanum is present in the composition of Ketac™ Molar (3M™ Espe), a glass ionomer cement from the same 3M™ ESPE Ketac™ product line designed for interproximal fillings and whose glass powder is composed of aluminum–calcium–lanthanum fluorosilicate glass; however, the company does not mention the presence of lanthanum in the composition of Ketac™ Cem (3M™ Espe).

As shown in Figure 5e,f, large silver carbonate aggregates can be observed with heterogeneous distribution, but the form in which these aggregates appear confirms that the silver carbonate is poorly distributed in the material, as they tend to aggregate.

Regarding the colorimetric evaluation, the incorporation of silver carbonate into the GIC leads to an important variation in the color of the samples, producing a notable reduction in their luminosity. This darkening of the samples with the incorporation of silver compounds into the GIC, acquiring a more reddish and yellowish tone compared to the control samples, agrees with the results of previously published research [18]. In contrast, the samples of GIC modified with inorganic glass with encapsulated silver did not show a remarkable color variation, only a slight decrease in brightness and a subtly more bluish tone, but these were practically imperceptible.

Based on the results of the present investigation, it is noteworthy that the modification of GIC by incorporating silver compounds could represent a potential way to improve glass ionomer cements due to their proven antibacterial and mechanical reinforcement effects. However, it would be interesting to extend the research and study the effect of ionomer modification in the long term under conditions similar to the oral cavity.

## 5. Limitations of the Study

For the color evaluation of the samples, a specialized instrument for the measurement of tooth color was used to measure the color of a material. However, this limitation was assumed since we only sought to determine the color variation to determine the possible differences or color changes.

Regarding the type of study and the follow-up carried out, the present investigation was a short-term study, with trials with short follow-up times. Since the purpose of this study was to determine which compound conferred better antibacterial results without sacrificing their physical and mechanical properties, a similar subsequent study is being considered to develop a more detailed study with longer follow-up times using the selected silver compounds. Another aspect to be evaluated in future investigations is the cytotoxicity of the selected silver compounds, which might be investigated by MTT assay, with the aim of assessing their biosafety to be able to use the modified materials in clinical practice. Additionally, a more detailed structural analysis of the different samples will also be carried out to determine if the incorporation of the different compounds in the native material alters its structural properties.

## 6. Conclusions

- The incorporation of silver compounds into glass ionomer cement improved its antibacterial capacity.
- Silver carbonate and inorganic glass with encapsulated silver were the compounds whose incorporation into glass ionomer cement resulted in a significant improvement in the antibacterial properties of the material.
- The incorporation of silver carbonate up to 1% and of inorganic glass with encapsulated silver up to 5% significantly improved the antibacterial capacity of the glass ionomer cement without compromising the shear bond strength.
- A slight variation in the color of the material was detected when silver carbonate compounds were incorporated, while this variation was not perceptible in the case of inorganic glass with encapsulated silver.
- The modification of the material did not lead to an alteration of the structure, although the silver structures were organized in heterogeneously distributed clusters.

## Figures and Tables

**Table 1:** Description of the composition of the groups included in this study.

	Powder Composition	Liquid Composition
B	Nonmodified glass powder	Nonmodified GIC liquid
SC0.5	Glass powder modified with 0.5% SC	Nonmodified GIC liquid
SC1	Glass powder modified with 1% SC	Nonmodified GIC liquid
SC2	Glass powder modified with 2% SC	Nonmodified GIC liquid
V1	Glass powder modified with 1% of encapsulated silver	Nonmodified GIC liquid
V2.5	Glass powder modified with 2.5% of encapsulated silver	Nonmodified GIC liquid
V5	Glass powder modified with 5% of encapsulated silver	Nonmodified GIC liquid
CH2.5	Nonmodified glass powder	GIC liquid modified with 2.5% CH
CH5	Nonmodified glass powder	GIC liquid modified with 5% CH
CH7.5	Nonmodified glass powder	GIC liquid modified with 7.5% CH
CH10	Nonmodified glass powder	GIC liquid modified with 10% CH
CH5-SC0.5	Glass powder modified with 0.5% SC	GIC liquid modified with 5% CH
CH5-SC1	Glass powder modified with 1% SC	GIC liquid modified with 5% CH
CH10-SC0.5	Glass powder modified with 0.5% SC	GIC liquid modified with 10% CH
CH10-SC1	Glass powder modified with 1% SC	GIC liquid modified with 10% CH

GIC: glass ionomer cement; CH: chitosan; SC: silver carbonate.

**Table 2:** Minimum bactericidal concentration of silver carbonate against *S. mutans* and *L. acidophilus*.

	*L. acidophilus*		*S. mutans*	
ppm SC	CFU/mL	% Reduction	CFU/mL	% Reduction
2000	0	100	0	100
1000	0	100	0	100
500	0	100	8.0 × 10^2^	99.9
250	0	100	9.7 × 10^2^	99.9
100	0	100	1.8 × 10^5^	91.9
50	0	100	9.6 × 10^5^	57.5
25	3.4 × 10^5^	85.4	1.1 × 10^6^	49.9
10	9.9 × 10^5^	57.3	1.6 × 10^6^	29.2

SC: silver carbonate; CFU: colony-forming units; mL: milliliters; ppm: parts per million.

**Table 3:** Shear test results expressed in MPa for each specimen in the three tests and the mean value of the three tests. The different superscripts (a–g) indicate statistically significant differences using the LSD test (*p* < 0.005).

Group	Shear Strength			
	Test 1 (MPa)	Test 2 (MPa)	Test 3 (MPa)	Mean (MPa)
B	8.68	8.22	8.97	8.62 ^g^
SC0.5	8.33	8.53	8.60	8.49 ^g^
SC1	7.92	8.14	8.37	8.14 ^fg^
SC2	7.99	8.01	7.73	7.91 ^f^
V1	7.82	9.24	8.39	8.48 ^g^
V2.5	8.80	8.36	7.82	8.33 ^fg^
V5	7.95	8.43	8.27	8.22 ^fg^
CH2.5	7.29	6.76	6.79	6.95 ^de^
CH5	6.58	6.85	6.81	6.75 ^d^
CH7.5	5.04	5.56	5.26	5.29 ^ab^
CH10	5.26	4.98	5.02	5.09 ^a^
CH5-SC0.5	7.08	7.34	7.59	7.34 ^e^
CH5-SC1	6.89	6.98	7.20	7.02 ^de^
CH10-SC0.5	6.19	5.96	6.08	6.08 ^c^
CH10-SC1	5.66	5.62	6.09	5.79 ^bc^

MPa: MegaPascal; B: nonmodified glass ionomer cement (GIC); C: positive control (cellulose impregnated with antibiotic); SC0.5: GIC modified with 0.5% SC; SC1: GIC modified with 1% SC; SC2: GIC modified with 2% SC; V1: GIC modified with 1% inorganic glass with encapsulated silver; V2.5: GIC modified with 2.5% inorganic glass with encapsulated silver; V5: GIC modified with 5% inorganic glass with encapsulated silver; CH2.5: GIC modified with 2.5% chitosan (CH); CH5: GIC modified with 5% CH; CH7.5: GIC modified with 7.5% CH; CH10: GIC modified with 10% CH; CH5-SC0.5: GIC modified with 5% CH and 0.5% SC; CH5-SC1: GIC modified with 5% CH and 1% SC; CH10-SC0.5: GIC modified with 10% CH and 0.5% SC; CH10-SC1: GIC modified with 10% CH and 1% SC.

**Table 4:** Multiple range test for MPa by material. Fisher’s least significant difference (LSD) method was used. There were no significant differences for those levels sharing the same X’s column.

Group	Cases	Mean	Homogeneous Groups
CH10	3	5.08667	X
CH7.5	3	5.28667	XX
CH10-SC1	3	5.79	XX
CH10-SC0.5	3	6.07667	X
CH5	3	6.74667	X
CH2.5	3	6.94667	XX
CH5-SC1	3	7.02333	XX
CH5-SC0.5	3	7.33667	X
SC2	3	7.91	X
SC1	3	8.14333	XX
V5	3	8.21667	XX
V2.5	3	8.32667	XX
V1	3	8.48333	X
SC0.5	3	8.48667	X
B	3	8.62333	X

B: nonmodified glass ionomer cement (GIC); C: positive control (cellulose impregnated with antibiotic); SC0.5: GIC modified with 0.5% SC; SC1: GIC modified with 1% SC; SC2: GIC modified with 2% SC; V1: GIC modified with 1% inorganic glass with encapsulated silver; V2.5: GIC modified with 2.5% inorganic glass with encapsulated silver; V5: GIC modified with 5% inorganic glass with encapsulated silver; CH2.5: GIC modified with 2.5% chitosan (CH); CH5: GIC modified with 5% CH; CH7.5: GIC modified with 7.5% CH; CH10: GIC modified with 10% CH; CH5-SC0.5: GIC modified with 5% CH and 0.5% SC; CH5-SC1: GIC modified with 5% CH and 1% SC; CH10-SC0.5: GIC modified with 10% CH and 0.5% SC; CH10-SC1: GIC modified with 10% CH and 1% SC.

**Table 5:** Values in the CIE L*a*b* color space resulting from the colorimetric characterization of the samples using VITA Easyshade^®^V.

Group	Color Characterization (cie L*a*b*)		
	L*	a*	b*
B	83.2	−0.7	36.6
SC0.5	66.1	2.2	28.9
SC1	56.1	5.2	23.8
SC2	52.4	5.4	23.2
V1	79.2	−0.1	31.2
V2.5	77.7	−0.1	31.4
V5	74.0	0.9	31.8
CH2.5	85.5	−1.3	38.9
CH5	82.8	−1.4	38.1
CH7.5	81.6	−0.9	35.4
CH10	79.9	−0.9	38.0
CH5-SC0.5	78.8	0.1	36.6
CH5-SC1	71.3	1.1	33.5
CH10-SC0.5	78.4	0.2	35.0
CH10-SC1	70.8	1.9	32.0

## Data Availability

The relevant data are contained within the article. Additional data are available from the corresponding author on reasonable request.
